# Curcumin-Functionalized Electrospun Nanofibrous Membranes with Antimicrobial Activity for Wound Healing

**DOI:** 10.3390/nano15050388

**Published:** 2025-03-03

**Authors:** Neraida Petrai, Konstantinos Loukelis, Maria Chatzinikolaidou

**Affiliations:** 1 Department of Materials Science and Engineering, University of Crete, 700 13 Heraklion, Greece; nerajdapet22@gmail.com (N.P.); loukelisk@yahoo.com (K.L.); 2 Foundation for Research and Technology Hellas (FORTH)-IESL, 700 13 Heraklion, Greece

**Keywords:** electrospinning, electrospraying, poly(vinyl alcohol) (PVA), kappa-carrageenan, wound dressing, antibacterial

## Abstract

Chronic or improperly healed wounds, either as a result of extended trauma or prolonged inflammatory response, affect a significant percentage of the world population. Hence, there is a growing interest in the development of biomimetic scaffolds that expedite wound closure at the early stages. Curcumin (Cur) is a plant-derived polyphenol with antimicrobial activity, and it accelerates the wound contraction rate. Recently, electrospraying has emerged for the precise deposition of bioactive molecules into scaffolds to improve therapeutic outcomes. In this study, we produced membranes for wound healing and endowed them with antibacterial properties to promote the healing of impaired wounds. Unlike previous studies that incorporated curcumin directly into electrospun fibers, we employed electrospraying to coat curcumin onto PVA/KC membranes. This approach improves the curcumin bioavailability and release kinetics, ensuring sustained therapeutic action. Toward this end, we fabricated four types of membranes, poly(vinyl alcohol) PVA and PVA/kappa carrageenan (KC), using electrospinning, and PVA/KC/Cur5 and PVA/KC/Cur20, in which the PVA/KC membranes were coated with two different concentrations of Cur by electrospraying. All membranes showed low cytotoxicity, good cell adhesion, the capability of enabling cells to produce collagen, and an adequate degradation rate for wound-healing applications. Antibacterial evaluation showed that both Cur-loaded membranes increased the antibacterial efficacy against both *Escherichia coli* and *Staphylococcus aureus* compared with PVA and PVA/KC membranes. These findings highlight the potential of electrosprayed curcumin as an effective strategy for bioactive wound dressings.

## 1. Introduction

Skin is the largest organ in the human body, providing the first line of defense against external damage but also serving as a wall barrier against pathogen infiltration. Moreover, it plays a key role in many vital functions, such as immunological surveillance, preventing loss of physiological fluids, synthesis of vitamin D3, and temperature regulation. After a skin injury, the immune system is activated to heal the wound quickly and prevent bacterial contamination. However, there are cases, usually seen in individuals suffering from serious illnesses such as diabetes or high levels of obesity, where the natural healing process is impaired, leading to the establishment of chronic wounds that can take a significant toll on the patient’s quality of life [1,2].

Recently, researchers have been considering using tissue-engineered scaffolds as wound-dressing agents that can mimic both the exterior and the mid inner layer of the skin, known as the epidermis and dermis, respectively. An ideal scaffold for wound healing, apart from supreme biocompatibility, should also possess an architectural structure and porosity that allow the exchange of nutrients but concurrently prevent the entry of harmful pathogens. In addition, the scaffold must be able to maintain its integrity but also slowly degrade in a fashion that would enable the gradual restoration of the targeted tissue [3].

Electrospinning is a technique that creates fibrous membranes using a high-voltage source that is capable of depositing them on a conductive collector. Due to the stochastic alignment of the fibers, electrospun membranes closely resemble the morphology of the natural extracellular matrix (ECM) mesh, which plays an essential structural and functional role in the wound-healing process. In addition, they exhibit high surface area properties, making them prime scaffolds for drug encapsulation and fluid absorption [4].

Electrospraying is based on the same fundamental principles as electrospinning. However, by manipulating particular parameters, the solvent evaporates more rapidly, leading instead to droplet sputtering. The dispersion of the liquid into droplets ensures precise delivery and deposition of substances, which plays a crucial role in their controlled release. With the progress of the electrospraying technique, new opportunities have emerged to deposit and encapsulate multiple substances such as polymers, cells, and bioactive molecules into scaffolds in a spatially controlled manner, and therefore, enhance their bioavailability. Thus, the combination of electrospinning and electrospraying opens up new possibilities for the development of scaffolds that are biomimetic, have antibacterial properties, and enable the controlled release of drugs [5].

Poly(vinyl alcohol) (PVA) is a highly hydrophilic synthetic polymer widely used in tissue engineering due to its biocompatibility and chemical resistance. In addition, scaffolds composed of PVA provide adequate mechanical stability, and its water solubility facilitates its use in biomedical applications [6]. However, the wide use of PVA as a scaffold for wound healing has been limited due to its low bioactivity and insufficient swelling. A method used to compensate for the drawbacks of PVA is blending it with various natural polysaccharides that enhance cell adhesion [7]. Carrageenans are water-soluble, linear, and natural polysaccharides extracted from red seaweeds. Kappa carrageenan (KC) in particular, has one sulfate group per disaccharide unit, which endows it with a negative charge at 7.4 pH [8]. KC has been widely used in the food industry due to its gel-forming ability, low cost, and non-toxicity. Recently, it has also gained attention in the skin tissue engineering field due to its biocompatibility, biodegradability, high fluid absorption and retention capacity, and bioactivity, as its structure resembles two major components of native extracellular matrices called endogenous glycosaminoglycans (GAGs), specifically chondroitin-4-sulfate and dermatan sulfate [8].

Curcumin (Cur) is a natural yellow polyphenolic substance extracted from the rhizome of *Curcuma longa*. Apart from its generalized use in the food industry, it has also found wide applicability in biomedical applications, as it has been reported to promote wound healing, mainly due to its anti-inflammatory properties and antibacterial activity [9]. In addition, Cur has antioxidant properties that may aid wound healing by preventing free radicals such as reactive oxygen species, which are present at high concentrations in chronic wounds. However, its high hydrophobicity and low bioavailability are two major drawbacks that limit its utilization at higher concentrations [10,11].

A recent study [12] reported on Cur-loaded PVA electrospun membranes and the Cur release from the PVA fibers as well as the antibacterial activity of the membrane against *S. aureus* and *E. coli* bacterial strains. They incorporated Cur into the PVA solution, which was then electrospun. The fibrous membranes showed high efficacy against both bacterial types, suggesting the potential of the electrospun membranes as wound dressings. However, the effect of the Cur-loaded membrane on cell viability and morphology was not investigated. Another report [13] describes an ampicillin-loaded hydrogel of PVA, KC, and hyaluronic acid that indicated antibacterial activity against *S. aureus* and *E. coli* and no cytotoxicity in the presence of L929 fibroblasts. However, the combination of PVA and KC for the fabrication of biomimetic electrospun nanofibrous membranes for wound healing has not been investigated. Although a Cur-loaded PVA membrane has been previously reported in the literature [14], this is the first time Cur particles are electrosprayed in a PVA/KC membrane. The main aim of this study was to fabricate nanofibrous biomimetic membranes using a natural polysaccharide (KC) and a synthetic hydrophilic polymer (PVA) and further functionalize them with Cur to endow them with antimicrobial properties. In contrast to previous reports [15] in which Cur was loaded onto scaffolds by direct incorporation into the solution, which was then electrospun, we report on electrospraying Cur onto the electrospun membranes.

Two of the main advantages of electrospraying Cur on the nanofibrous membranes constitute the improved aqueous solubility of the poorly water-soluble Cur as well as its enhanced bioavailability. In particular, electrospraying Cur directly onto the surface of the nanofibrous membranes may improve its release kinetics compared with encapsulating it within the polymer fiber matrix. Based on other research studies reporting the release mechanisms of drugs from nanofibrous membranes [16,17], we assumed that in electrosprayed Cur-coated membranes, the Cur molecules are located in the outer layers of the nanofibrous membrane. Thus, there are fewer layers through which Cur diffuses, making the drug more accessible to the wound area. Moreover, the hydrophobic nature of Cur makes its mixing with water-soluble compounds rather challenging. Electrospraying overcomes this issue, as the electrospun membrane is prepared separately, and Cur is dissolved in a different solvent and electrosprayed into the membrane in a second step. Hence, we fabricated biomimetic electrospun nanofibrous membranes from PVA and KC and coated them with Cur to enhance them with antibacterial properties. Our hypothesis was to investigate the effect of two different Cur concentrations by adjusting the duration of electrospraying for the functionalization of the PVA/KC membranes with Cur on the cellular responses in fibroblasts and bacteria. To this end, we fabricated four types of electrospun nanofibrous membranes, PVA and PVA/KC via electrospinning and PVA/KC/Cur5 and PVA/KC/Cur20, in which a Cur solution was electrosprayed onto the PVA/KC membranes for 5 and 20 min, respectively. The physicochemical properties of the membranes and the release kinetics of Cur were investigated. L929 fibroblasts were cultured on fibrous scaffolds, and the cell migration along with the cell viability, cell morphology, and collagen secretion were evaluated. In addition, the antibacterial activity of the Cur-functionalized membranes against Gram-positive *S. aureus* and Gram-negative *E. coli* bacteria was investigated.

## 2. Materials and Methods

### 2.1. Materials

Polyvinyl alcohol (Mw 145,000 kg/mol), k-carrageenan, and curcumin (from *Curcuma longa*), were purchased from Sigma-Aldrich (St. Louis, MO, USA). The Presto Blue^TM^ reagent was purchased from Invitrogen Life Technologies (Carlsbad, CA, USA). Trypsin/EDTA (0.25%) and phosphate buffer saline (PBS) were purchased from Gibco ThermoFisher Scientific (Waltham, MA, USA). Span^®^ 80 solution, hexamethyldisilizane (HMDS), paraformaldehyde (PFA), and Sirius Red 90 were purchased from Sigma-Aldrich (St. Louis, MO, USA). Penicillin/streptomycin (P/S) was purchased from Gibco ThermoFisher Scientific (Waltham, MA, USA). Roswell Park Memorial Institute (RPMI) medium and fetal bovine serum (FBS) were purchased from PAN-Biotech (Aidenbach, Germany). Augmentin was purchased from GlaxoSmithKline. Mueller–Hinton broth (MH-B) was purchased from Sigma-Aldrich (St. Louis, MO, USA).

### 2.2. Preparation of Electrospinning and Electrospraying Solutions

The PVA solution for electrospinning was prepared by dissolving 5% *w*/*v* PVA in distilled water. The mixture was stirred continuously with a magnetic stirrer for 3 h at 90 °C and 250 rpm to ensure complete dissolution. To produce PVA/KC membranes, the solution was obtained by preparing the PVA solution in the same manner and subsequently adding 1% *w*/*v* KC. The mixture was maintained under stirring for 4 h at 70 °C and 250 rpm. Moreover, 25 μL of Span 80 detergent was added to the mixture to lower the surface tension and facilitate the electrospinning process. To produce Cur-loaded membranes, another solution was prepared by dissolving 10% *w*/*v* Cur in 1:1 volume ratio of dimethyl sulfoxide (DMSO) and water solvent. Initially, 0.1 g of Cur was added to 0.5 mL of dimethyl sulfoxide, and after Cur was completely dissolved, 0.5 mL of water was added in a controlled manner. The solution was maintained under gentle stirring at room temperature for about 3 h.

### 2.3. Electrospinning and Electrospraying Process

The scaffolds used in this work were fabricated by means of a custom-made electrospinning device by CommonsLab SCE (Heraklion, Greece), similar to previous reports using a blend of PVA and a polysaccharide [18]. The electrospinning method includes the following essential elements: a polymer solution, a horizontal syringe plunger controlled by a console, a blunt needle, a flat collector covered with aluminum foil, and a high-voltage supply with a range of 0–30 kV. A high-voltage source is applied after a polymer solution is loaded into the syringe. When the electrostatic interactions overcome the surface tension, the Taylor cone is formed, creating an accelerated jet deposited on the collector. To produce PVA and PVA/KC fibrous membranes, a total of 10 mL of polymer solution was loaded into a plastic syringe. The electrospinning parameters were set as follows: (i) flow rate at 1 mL/h, (ii) distance between the blunt needle and the grounded collector at 15 cm, (iii) applied voltage at 14 and 13 kV for the PVA and PVA/KC membranes, respectively. These parameters were selected based on previous work in the group. Various conditions were assessed to achieve stable jet formation and uniform fiber morphology, and the final conditions were selected based on the most consistent results. Although we maintained a stable processing environment, minor variations in humidity and temperature may have contributed to differences in fiber diameter and uniformity.

For the fabrication of the Cur-loaded scaffolds, the same electrospinning setup was employed, but this time the purpose was to generate Cur-loaded droplets instead of fibers via electrospraying. After 1 mL of the Cur solution was loaded into a 5 mL syringe, the distance was set at 20 cm, the flow rate at 1 mL/h, and the applied voltage at 21 kV. The Cur-loaded droplets were deposited onto the PVA/KC membranes. Two different types of PVA/KC/Cur membranes were produced; in one type, the Cur electrospraying process lasted 5 min, and in the other type, 20 min. Table 1 summarizes the membrane types. After production, the fibrous membranes were removed from the aluminum foil and stored appropriately until use.

### 2.4. Physicochemical Characterization of the Electrospun Membranes

#### 2.4.1. Fourier Transform Infrared Spectroscopy (FTIR)

The infrared spectrum of absorption for the fibrous scaffolds was measured by a Nicolet 6700 optical spectrometer within the range of 4000–400 cm^−1^. Measurements were performed using three samples (n = 3), and the data collected were transferred to Origin software 10.1 for graphical representation.

#### 2.4.2. TGA

The thermal stability of the electrospun scaffolds was assessed by means of the thermogravimetric (TG) analysis using a TGA5500 thermogravimetric analyzer (TA instruments, New Castle, DE, USA). The samples were placed into a platinum pan, and their weight was recorded from 25 °C to 900 °C at a heating rate of 10 °C/min. The analysis was performed using three samples (n = 3) for each membrane composition.

#### 2.4.3. Contact Angle Measurement

The hydrophilicity of the fibrous membranes was measured with a contact angle computing device (OCA 15 Plus, Data Physics Instruments, Filderstadt, Germany). Distilled, deionized water droplets with a volume of 4 μL were dripped onto the samples from a motorized syringe at ambient temperature and pressure. Measurements were taken at four different positions for each sample, and the average of four replicates per membrane (n = 4) was reported to ensure reproducibility.

#### 2.4.4. Swelling and Degradation Profile

To determine the swelling capability of the scaffolds, the fibrous membranes were cut into identical shapes. After their weight was measured, they were placed into 24-well plates. They were then immersed in phosphate-buffer saline (PBS) with a pH of 7.4. At predetermined time points (1 and 24 h), the buffer was aspirated, and the excess water was carefully removed from the membrane with a paper towel. After allowing the membranes to dry for 1 h, their weight was measured again. The degree of swelling of the fibrous scaffolds was calculated using the following formula:

$$swelling\;ratio\;\%=\frac{Wwet-Wdry}{Wdry}*\times 100\;$$

where W_dry_ is the weight of scaffolds before and W_wet_ is the weight after they were immersed in the buffer.

The same process as before was used to determine the degradation rate of the scaffolds, but this time the plate was placed in an incubator (Thermo Scientific™, Forma™ Steri-Cycle™ CO_2_ Incubator, Waltham, MA, USA) at 37 °C after immersion in phosphate-buffer saline (PBS). After 7, 14, and 21 days, their weight was measured. A high-precision scale (Analytical balance ABT 220-5DNM) with an accuracy of ±1 μg was used for the weight measurements.

### 2.5. Curcumin Release

The Cur release profile experiment was conducted to determine the amount of Cur released from the fiber mats. First, 0.6 × 0.6 cm Cur-loaded samples were prepared and placed in 48-well plates. The samples were then immersed in phosphate-buffer saline (PBS) mixed with 0.2% *v*/*v* tween 20. Tween 20 was added to PBS due to Cur’s insolubility in water. After the plate was incubated at 37 °C, the supernatants were collected and replaced with an equal volume of fresh PBS medium at specific time points. The amount of Cur in each collected solution was analyzed using a UV-Vis spectrophotometer at 421 nm. A calibration curve of Cur in the same buffer was used to convert the obtained absorbance values to Cur concentration. The accumulative weight of Cur over time was plotted as a function of incubation time to visualize the release kinetics.

### 2.6. Preparation of Scaffolds for the Fibroblasts Cell Culture

Before the cell seeding, all scaffolds were sterilized with UV light for 15 min on each side. They were then cut into squares of equal size and placed into 48-well plates. Afterward, they were immersed in 250 μL of cell culture medium for 1 h. The medium was then aspirated, and the membranes were allowed to dry for 2 h before cell seeding.

### 2.7. Scaffold Morphology and Cell Adhesion

To investigate cell attachment and proliferation on the different scaffolds, the membranes were visualized using scanning electron microscopy (SEM) (JEOL JSM-6390 LV, Tokyo, Japan). The cell-cultured plate was maintained in an incubator at 37 °C at 0.5% CO_2_. On days 3 and 7, the medium was carefully aspirated from the 48-well plate, and the samples were fixed in 4% *v*/*v* paraformaldehyde for 25 min. They were then washed with phosphate-buffer saline (PBS) and dehydrated with a series of increasing ethanol solutions (from 10 to 100% *v*/*v*). Afterward, they were immersed in hexamethyldisilazane for 30 min, and after being air-dried overnight, they were sputter-coated with gold (Baltec SCD 050, Pfäffikon, Switzerland).

### 2.8. Cell Viability on the Fibrous Scaffolds

Presto Blue^TM^ Reagent, a resazurin-based solution, was used to study the viability and growth of cells on the fibrous membranes. After adding Presto Blue^TM^ to the cells, resazurin is reduced to resorufin, a highly fluorescent compound, due to cell metabolism. The color changes are detected by the absorbance measurements. For the cell viability study, L929 fibroblast cells were seeded at a density of 7 × 10^4^ cells/well in a 48-well plate. The number of cells per well was measured after 2, 5, and 7 days. Three independent experiments were conducted, each in triplicates per condition. The medium was aspirated, and 180 μL of fresh medium was pipetted along with 20 μL of Presto blue. The plate was then incubated for 1 h at 37 °C and 5% CO_2_. Subsequently, 100 μL from each well was transferred to a 96-well plate, and the absorbance of the contents of each well was measured at 570 and 600 in a spectrophotometer (Synergy HTX Multi-Mode Micro-plate Reader, BioTek, Bad Friedrichshall, Germany).

### 2.9. Determination of the Secreted Collagen

The Sirius Red Dye assay was used to determine collagen secretion from cells cultured on the four membrane compositions compared with the TCPS control. The negatively charged sulfonic acid groups present in the dye bind ionically to the positively charged amino groups present in the collagen species produced by the cells [18]. After 3 × 10^4^ fibroblasts were seeded into each membrane in a 48-well plate, the plate was incubated at 37 °C for 21 days. The supernatants were collected and replaced with fresh medium after 7, 14, and 21 days. At a subsequent time, when all the supernatants from all the time points were collected, 25 μL supernatants of each sample were transferred in 1.5 mL Eppendorf tubes and diluted in dH_2_O to a final volume of 100 μL. To isolate the collagen precipitate, 500 μL of 0.1% *w*/*v* Sirius Red Dye solution in 0.5 M acetic acid was added to all tubes and centrifuged at 4 °C for 30 min at a speed of 15,000× *g*. To remove the excess dye from the collagen pellet, the supernatants were then removed, and after 1 mL of 0.5 M acetic acid was added, the Eppendorf tubes were centrifuged for 10 min. This procedure was repeated several times, until the supernatants were transparent. Finally, the collagen pellet was diluted with 500 μL of 0.1 N NaOH, and 100 μL of the resulting solutions was transferred to a 96-well plate. The plate was placed in a spectrophotometer, and the absorbance values at 530 nm were recorded. The absorbance values were converted to collagen concentration in μg/mL using a standard collagen curve. Three independent experiments were conducted, each in triplicates per condition.

### 2.10. Cell Migration Assay

To evaluate the impact of Cur on cell migration, an in vitro wound-healing scratch assay was employed. Initially, the Cur released from pre-sterilized PVA/KC/Cur5 and PVA/KC/Cur20 scaffolds was collected. After adding 1 mL RPMI to each scaffold, the plate was incubated at 37 °C. After 48 h, the supernatants were carefully collected in Eppendorf tubes and stored appropriately until use.

At the same time, L929 cells were cultured in RPMI, and when they were 80% confluent, they were seeded in a 96-well plate at a density of 4 × 10^4^ cells/well and incubated at 37 °C. When they reached confluency, the medium was aspirated, and a scratch was made mechanically in each well using a 100 μL tip. Subsequently, the wells were treated with three different media: pure RPMI medium (control) and the Cur supernatants collected earlier from the release of PVA/KC/Cur5 and PVA/KC/Cur20 scaffolds. The progress of cell migration was visualized under the microscope at 0, 24, and 48 h. The wound gap at the predetermined time points was measured using ImageJ software 1.54g with the assistance of the wound healing size tool. Three independent experiments were conducted, each with three samples (n = 3) per condition. The formula used for the calculation of the wound healing for each time point was

$$Wound\;closure\;\%=\frac{AreaT0-AreaTf}{AreaT0}*100.$$

### 2.11. Antibacterial Assays

#### 2.11.1. Preparation of the Bacterial Suspension

In this study, Gram-negative *E. coli* and Gram-positive *S. aureus* were used to determine the ability of Cur-loaded scaffolds to inhibit bacterial action. The strains have been described previously [19]. The bacterial strains were inoculated into 2.5 mL Mueller–Hinton Broth (MH-B) standard media. The bacterial suspensions were then maintained in a shaking incubator at 37 °C at 250 rpm for approximately 20 h. To assess the cell number, the suspensions were diluted in MH-B, and the corresponding concentrations were determined by acquiring the optical density (OD) values using a UV-Vis spectrophotometer (Synergy HTX Multi-Mode Micro-plate Reader). The OD 0.1 at 600 nm, corresponding to 2 × 10^7^ colony-forming units (CFU) per mL, was used for normalization of the bacteria cell suspension. For each experiment, after normalization of the bacteria suspension, the appropriate concentration was acquired through serial dilutions. The bacterial strains were stored as glycerol stocks at −80 °C.

#### 2.11.2. Bacterial Cell Viability Assessment

The membranes were cut into equal shapes, and after being sterilized for 30 min on each side, they were placed on a 48-well plate. After serial dilutions, each bacterial suspension was normalized to an OD of 0.1 that corresponds to 2 × 10^7^ CFU/mL, and bacterial suspension of 2 × 10^5^ CFU/mL was obtained through dilution. Subsequently, 50 μL of the acquired bacterial suspension was seeded in each well of the 48-well plate, and media was supplemented at a final volume of 200 μL. The bacteria viability was determined after 3 and 24 h using the Presto Blue^TM^ reagent in a ratio of 1:10. Subsequently, 100 μL from each well was transferred to a 96-well plate, and the OD values were recorded at 570 and 600 nm. Augmentin at a concentration of 100 μg/mL was used as positive control.

#### 2.11.3. Morphological Observation by SEM

The membranes were cut into equal shapes and placed in a 48-well plate. After obtaining a bacterial suspension of 2 × 10^5^ for both bacterial species, 50 μL of the suspensions was seeded into each sample, and an additional 150 μL of MH broth was subsequently added. The plate was then incubated at 37 °C, and after 4 and 24 h, the medium was carefully aspirated from the 48-well plate, and the samples were fixed in 4% *v*/*v* paraformaldehyde for 25 min. They were then washed with phosphate-buffer saline (PBS) and dehydrated with a series of increasing ethanol solutions from 10 to 100% *v*/*v*. They were then immersed in hexamethyldisilazane for 30 min and sputter-coated with gold (Baltec SCD 050) after overnight air-drying. The morphology of *E. coli* and *S. aureus* on the fibrous scaffolds was observed by a scanning electron microscope (SEM) as previously described [20].

#### 2.11.4. Kinetic Study

Initially, the supernatants of Cur release from the PVA/KC/Cur5 and PVA/KC/Cur20 scaffolds were obtained at specific time points. Initially, the Cur-loaded membranes were sterilized with UV radiation and were cut into identical shapes. They were then placed in 1.5 mL Eppendorf tubes, and after 500 μL of MH-B media was pipetted into each Eppendorf tube, they were maintained in a shaking incubator at 37 °C at 250 rpm. At predetermined time points (2, 6, 24 h), 150 μL of the medium was collected from each tube and was stored appropriately until use.

For the kinetic study, a 50 μL bacterial suspension of 2 × 10^5^ CFU/mL was added to a 96-well plate [20]. Subsequently, the Cur release media collected previously from the fibrous membranes was added to each well. The bacterial suspension in pure medium was used as negative control, and bacterial suspension in medium with 100 μg/mL augmentin was used as positive control. The plate was then transferred to a plate reader spectrophotometer at 37 °C, and the optical density values were measured at 600 nm every 15 min for 24 h.

### 2.12. Statistical Analysis

The statistical analysis of the experimental data was conducted with GraphPad Prism version 8 software. A two-way ANOVA (or mixed model) followed by Tukey’s multiple comparisons test was used to evaluate the significance between the mean values of the various scaffold compositions. A *p*-value < 0.05 was considered significant.

## 3. Results

### 3.1. Physicochemical Characterization

#### 3.1.1. Morphological Properties of Nanofibrous Membranes

**Figure 1:**
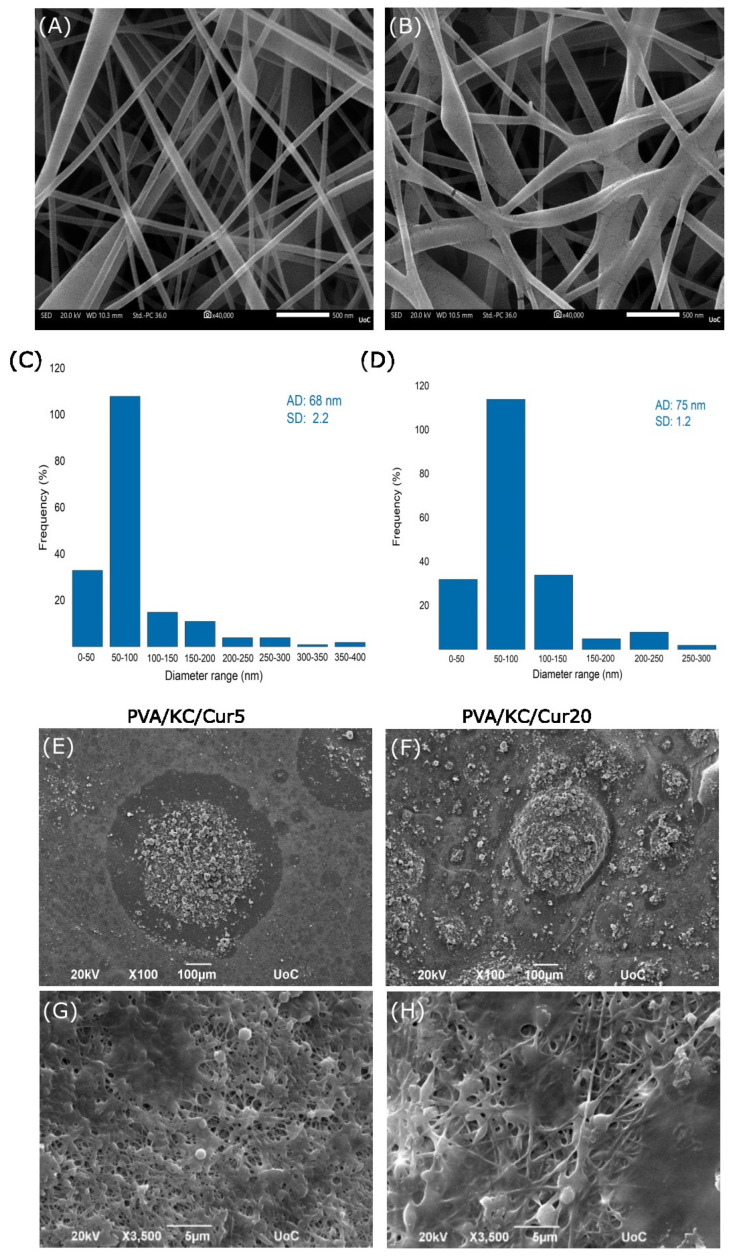
Representative SEM images at a magnification of 40,000 of (**A**) PVA and (**B**) PVA/KC. Scale bar represents 500 nm. Diameter distribution of (**C**) PVA and (**D**) PVA/KC fibrous membranes (AD: average diameter; SD: standard deviation). SEM imaging of PVA/KC/Cur5 (**E**,**G**) and PVA/KC/Cur20 (**F**,**H**) at two different magnifications (×100 and ×3500). The higher-magnification images (**G**,**H**) depict the fibrillar structure of the electrospun membranes beneath the electrosprayed curcumin.

The morphological properties and fiber diameters of the developed electrospun membranes are determined by the process parameters, the viscosity of the solution, and the surface tension. Kappa-carrageenan is known for its high thickening properties. Therefore, a series of experiments was carried out with different solutions with a PVA concentration of 5% *w*/*v* and a KC concentration varying from 0.5% to 1.5% *w*/*v*. The results showed that due to the highly viscous nature of KC, electrospun nanofibrous membranes could not be formulated above the threshold of 1% because the electrospinning process was unstable, with an intense elongation of the Taylor cone. As a result, the solution with a concentration of 1% *w*/*v* KC was selected for our experiments. However, the viscous nature of the solution complicated the electrospinning process. To facilitate electrospinning, the biocompatible and cytocompatible non-ionic surfactant sorbitan monooleate (Span 80) was integrated into the PVA/KC solution to reduce its viscosity. Detergents are able to facilitate the electrospinning process by breaking the bonds of the water molecules and subsequently reducing the surface tension. Figure 1A,B show the morphology of the PVA and PVA/KC membranes using SEM imaging, while the plots of fiber diameter distribution for each membrane are presented in Figure 1C,D. As shown in the images, both membranes had a similar average fiber diameter, ranging from 68 to 75 nm. The average diameter was 68 ± 2.2 nm for PVA and 75 ± 1.2 nm for PVA/KC.

We examined two Cur-loaded membranes using SEM imaging at two magnifications. Figure 1E,F depict the overall distribution of Cur particles on the membrane surfaces, while the high-magnification images (Figure 1G,H) show the spherical Cur particles embedded in the nanofiber network, with the nanofibers still visible beneath these particles. Comparing the two membranes, PVA/KC/Cur20 shows a significantly higher density of Cur particles on the surface, indicating that a longer deposition time leads to a higher Cur loading.

#### 3.1.2. FTIR Analysis

**Figure 2:**
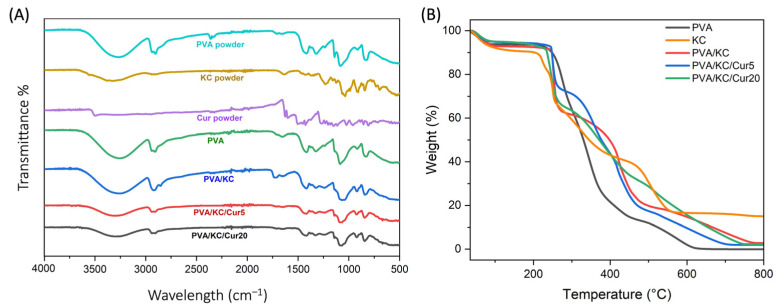
Graphical representation of (**A**) Fourier transform infrared spectra of the ingredients in powder form (PVA, KC, and Cur) and the four compositions, PVA, PVA/KC, PVA/KC/Cur5, and PVA/KC/Cur20, and (**B**) thermal gravimetric analysis (TGA) of the four compositions.

The FTIR spectra of PVA, KC, Cur, and the composite electrospun membranes PVA, PVA/KC, PVA/KC/Cur5, and PVA/KC/Cur20 are shown in Figure 2A. The PVA powder and PVA membrane exhibited almost identical spectra. The broad absorption peak at 3262 cm^−1^ results from a stretching vibration due to OH- groups. Two peaks at 2942 and 2907 cm^−1^ indicate the asymmetric and symmetric stretching of the CH_2_ group, respectively. Finally, the peaks at 1322 and 1080 cm^−1^ are attributed to the presence of C-O bonds [21]. Pure KC exhibits a series of peaks that are characteristic of its chemical structure. The peak at 1235 is representative of the symmetric vibration of the O=S=O group present on its sulfate groups, while the peak at 840 is attributed to the stretching vibration of -O-SO_3_ [22]. Moreover, the broad peak at 3500–3000 is due to the -OH groups present on the disaccharide [8]. The pure Cur also exhibited peaks corresponding to the vibrations of its functional groups. The absorption peaks of Cur at 3501, 1599, 1508, 1274, and 1152 cm^−1^ result from the vibration of the benzene ring, C=C vibrations, C-O stretching, and C-O-C stretching, respectively [23]. The PVA/KC membrane exhibited a spectrum similar to that of the PVA membrane, with minor differences attributed to interactions between PVA and KC. In the PVA spectrum, a small peak was observed at 1630 cm^−1^. In the PVA/KC spectrum, the increased intensity of this peak indicates the presence of KC within the membrane structure [19]. Additionally, the peaks in the PVA membrane at 2907 cm^−1^ (CH_2_ stretching) and 1080 cm^−1^ (C–O bonds) appear smoother in the PVA/KC membrane. The peak at 1322 cm^−1^, also indicating C–O bonds, is less prominent in the PVA/KC membrane. This smoothing and reduction in prominence of these peaks suggests interactions between PVA and KC. The spectra of PVA/KC/Cur5 and PVA/KC/Cur20 were nearly identical to each other and resembled that of the PVA/KC membrane. However, a notable difference was observed in the broad absorption peak at 3262 cm^−1^, corresponding to -OH groups, which was significantly less pronounced in the PVA/KC/Cur membranes. This reduction suggests an interaction between Cur and the PVA/KC membrane.

#### 3.1.3. TGA

The TGA technique was employed to investigate the thermal behavior of the different scaffold compositions, and their mass loss was recorded as a function of temperature (Figure 2B). An initial 4% weight loss between 35 and 100 °C was observed for all the scaffold compositions. This might be due to an initial loss of residual moisture and solute from the membranes. The decomposition of the PVA membrane mainly took place from 300 to 430 °C, where it lost almost 78% of its mass. This weight loss can be attributed to the decomposition of the side chain of PVA. The third smaller step, which takes place at 430 °C, indicates the decomposition of the main chain of PVA. These results are in agreement with data reported in other research papers [24]. The TGA analysis of the other membranes also revealed three distinct zones of mass loss. For the PVA/KC membrane, following the initial water loss, the major degradation occurred between 270 °C and 450 °C. A third degradation stage was observed between 470 °C and 600 °C. In the Cur-loaded membranes, the main decomposition phase shifted to a higher temperature range of 300 °C to 500 °C, followed by an additional stage between 500 °C and 700 °C.

#### 3.1.4. Contact Angle Measurement

**Figure 3:**
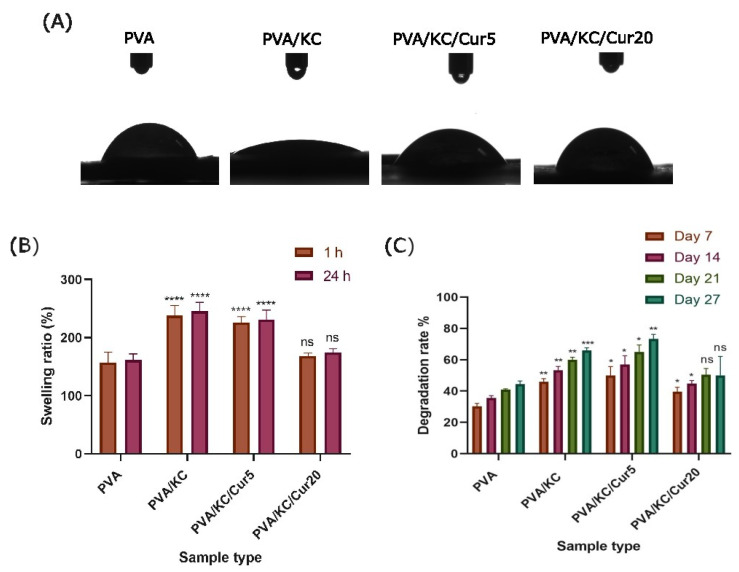
Graphical representation of (**A**) the contact angle, (**B**) the swelling ratio after 1 and 24 h in PBS 27 (statistical analysis was performed with ns > 0.05, **** *p* < 0.0001 compared with the PVA membrane), and (**C**) the degradation rates after 7, 14, 21, and 27 days (statistical analysis was performed with non-statistical differences (ns) > 0.05, * *p* < 0.05, ** *p* < 0.01, *** *p* < 0.001 compared with the PVA membrane) of the four scaffold compositions PVA, PVA/KC, PVA/KC/Cur5, and PVA/KC/Cur20. Each bar represents the mean ± SD of triplicates.

The hydrophilic properties of the fibrous membranes were evaluated with a contact angle measurement. Figure 3A shows the contact angles of the four scaffold compositions. All membranes exhibited a contact angle of less than 90°, demonstrating the hydrophilicity of all samples. The contact angle values were 63 ± 3°, 16 ± 1°, 46 ± 2°, and 64 ± 1° for the PVA, PVA/KC, PVA/KC/Cur5, and PVA/KC/Cur20 membranes, respectively. The PVA and PVA/KC/Cur20 composites had the highest contact angles. The PVA/KC membrane exhibited the lowest contact angle, and therefore, better hydrophilic properties. Electrospraying Cur onto the PVA/KC membrane increased the contact angle with increasing Cur concentration.

#### 3.1.5. Swelling and Degradation

The swelling ratio of the different scaffold compositions was recorded after 1 and 24 h in PBS. All samples exceeded their dry weight after 1 and 24 h at PBS. As illustrated in Figure 3B, after 1 h the swelling ratios were 157 ± 18%, 238 ± 17%, 226 ± 10%, and 169 ± 5% for PVA, PVA/KC, PVA/KC/Cur5, and PVA/KC/Cur20, respectively. After 24 h, only a slight increase in the swelling ratio was observed compared with the swelling ratio at 1 h, but this was not statistically significant. The PVA/KC fibrous membranes exhibited a greater capacity for swelling than the PVA membrane. The PVA/KC/Cur5 membrane had a slightly lower swelling capacity than the PVA/KC membrane, but the difference was not significant. However, the swelling ratio decreased significantly for the PVA/KC/Cur20 membrane.

The evaluation of the degradation rate was also investigated after 7, 14, 21, and 27 days at 37 °C to resemble the physiological conditions of the skin tissue (Figure 3C). The findings of this investigation demonstrated that all the fibrous membranes exhibited a gradual increase in their degradation rates over time. The PVA/KC membranes had the highest degradation rate among the scaffold compositions for all the days. The results demonstrated that after the electrospinning of Cur into the PVA/KC membranes, the degradation rate decreased for both membranes, and for the PVA/KC/Cur20, the decrease was more significant at days 21 and 27.

### 3.2. Curcumin Release Rate

**Figure 4:**
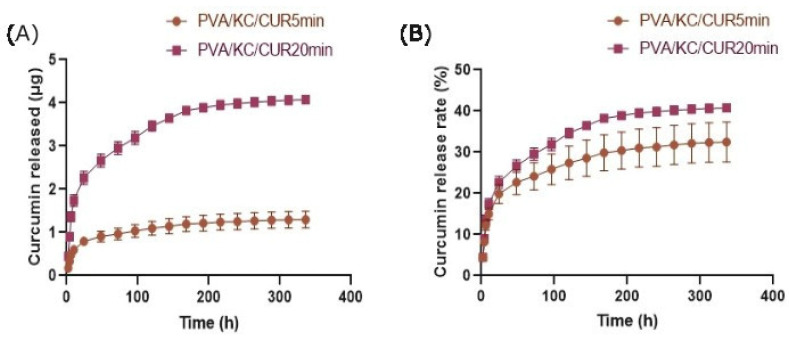
Graphical representation of Cur release from PVA/KC/Cur5 and PVA/KC/Cur20 scaffolds: released mass (**A**) and % release rate (**B**).

The Cur release rate from the PVA/KC/Cur5 and PVA/KC/Cur20 scaffolds was measured and recorded for 14 days (Figure 4). The release of Cur from the PVA/KC/Cur20 electrospun membrane was significantly higher than that of the PVA/KC/Cur5. The Cur released after 14 days was approximately 4.5 μg and 2 μg for the PVA/KC/Cur5 and PVA/KC/Cur20 membrane, respectively (Figure 4A). Initially, a burst release of Cur was observed in both membranes, followed by a gradual release that eventually reached a constant value at the end of the experiment.

### 3.3. Cell Adhesion and Morphology

**Figure 5:**
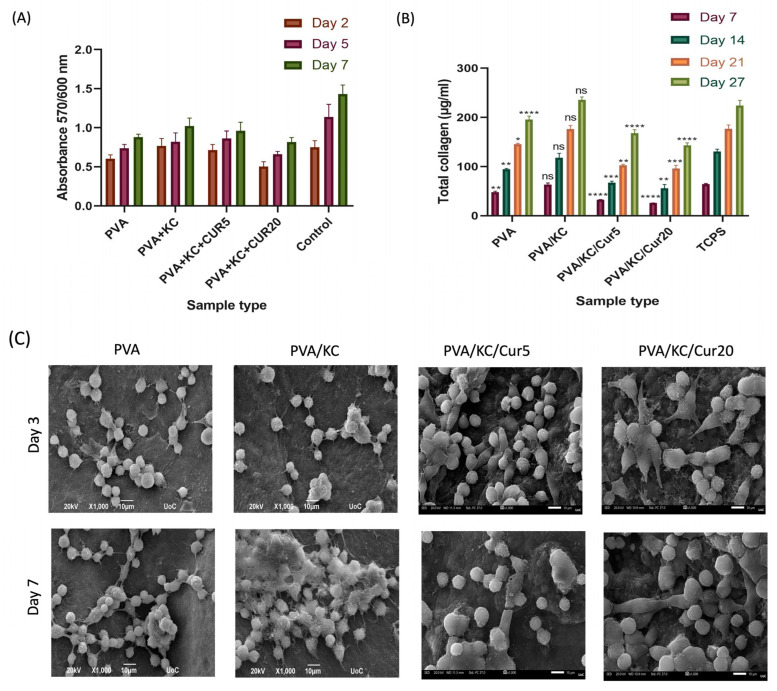
(**A**) Cell viability and proliferation of fibroblasts showing the absorbance at 570/600 of L929 cells on days 2, 5, 7; (**B**) collagen concentration at days 7, 14, 21, and 27 (statistical analysis with non-significant (ns) *p* > 0.05, * *p* < 0.05, ** *p* < 0.01, *** *p* < 0.001, **** *p* < 0.0001 compared with the TCPS control); and (**C**) SEM images showing the morphology of L929 cells after 3 and 7 days in culture seeded into the PVA, PVA/KC, PVA/KC/Cur5, and PVA/KC/Cur20 nanofibrous membranes. Values are expressed as mean ± standard deviation. Scale bars represent 10 μm.

The morphology of L929 fibroblasts seeded on the different scaffold compositions was observed via SEM on days 3 and 7 (Figure 5C). All electrospun composite scaffolds exhibited a strong adhesion profile on both days. The SEM images demonstrate that some of the cells have assumed an elongated shape on both days, and even though some round cells that have not yet adhered to the membranes are present, the absence of the electrospun fibers in the SEM images indicates that thick cell layers that completely cover the surface of the membranes are formed.

### 3.4. Cell Viability and Proliferation

To investigate the potential use of the scaffolds of this study in wound healing, the cytotoxicity of the electrospun membranes was investigated by means of the PrestoBlue^®^ cell viability assay. Figure 5A shows the absorbance values of all the composite fibrous membranes compared with the TCPS control. It was found that none of the composite scaffolds exhibited any cytotoxic effect over the course of 7 days. The absorbance values of the L929 cells on the PVA/KC membranes are higher than on the PVA membranes, indicating that the inclusion of KC enhanced the biocompatibility of the membrane on all days of the study. The PVA/KC membrane and the TCPS control had relatively close absorbance values on days 2 and 5. There was no significant difference in absorption between the PVA/KC and PVA/KC/Cur5 membranes for all days. However, there was a significant decrease in absorbance values for the PVA/KC/Cur20 membrane compared with PVA/KC and PVA/KC/Cur5. The cell viability results are in line with the findings from SEM analysis.

### 3.5. Collagen Secretion

The collagen produced by the cells cultured on the electrospun membranes was investigated over a period of 27 days (Figure 5B). The collagen concentration increased in all samples at all time points. The cells seeded on the PVA/KC membrane had the highest collagen production levels at all time points, which was comparable to the TCPS control. The cells seeded on PVA/KC/Cur5 and PVA/KC/Cur20 membranes had the lowest collagen concentration. The collagen secreted by the cells seeded on the PVA/KC membrane was higher than that of the cells seeded on the PVA membrane. However, the collagen concentration decreased in the PVA/KC/Cur5 and PVA/KC/Cur20 membranes compared with the PVA/KC membrane on all days.

### 3.6. Cell Migration

**Figure 6:**
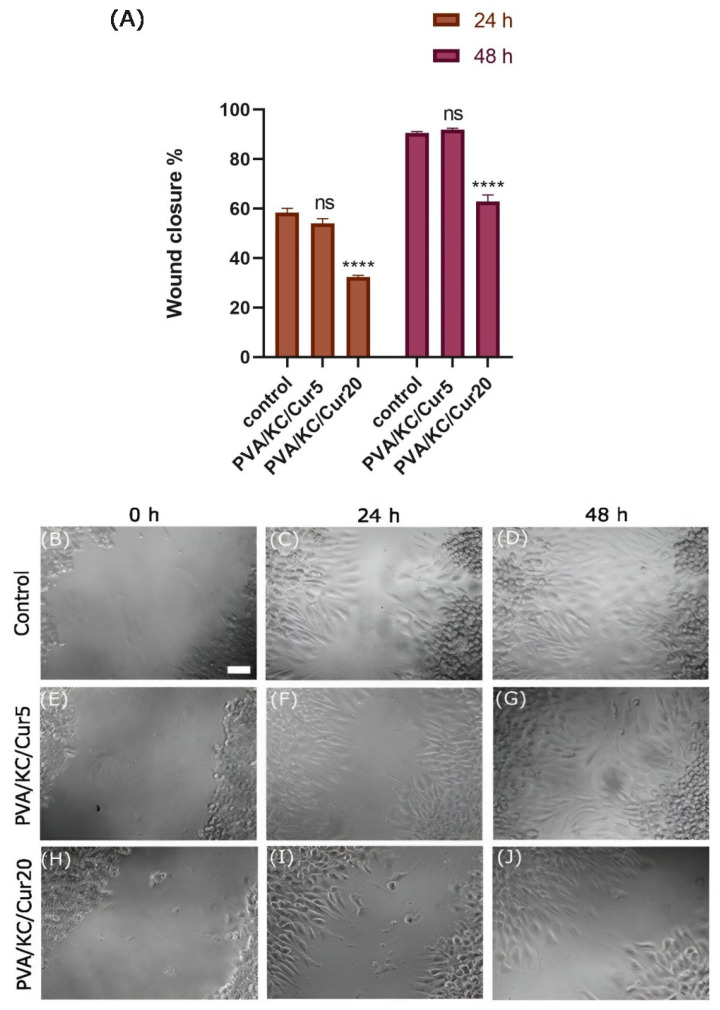
(**A**) Graphical representation of wound healing. (**B**–**J**) Images of cell migration of L929 cells after 0, 24, and 48 h following induction in the releasate of the PVA/KC/Cur5 and PVA/KC/Cur20 membranes. Control is the condition where TCPS was treated with pure medium. Scale bar represents 40 μm. Statistical analysis was performed in comparison with the cell migration induced in pure culture medium (ns *p* > 0.05 and **** *p* < 0.0001).

The migration of the L929 fibroblasts in the presence of the Cur-loaded scaffolds’ releasate was investigated using an in vitro cell migration assay (Figure 6). After 24 h, wound healing was 58 ± 8% for the cells treated with normal RPMI medium (control group) and 54 ± 8% and 32 ± 7% for the PVA/KC/Cur5 and PVA/KC/Cur20 releasates in RPMI cell culture medium, respectively. After 48 h, wound healing reached 91 ± 8%, 92 ± 9%, and 63 ± 10%, respectively (Figure 6A). There was no significant difference in wound healing at 24 and 48 h between the control and PVA/KC/Cur5 groups. Therefore, the PVA/KC/Cur5 scaffolds did not impair the normal migration rate of L929 cells in vitro. However, the migration rate of L929 cells was significantly slower in the cells treated with PVA/KC/Cur20 releasate than in the cells cultured with normal RPMI and with PVA/KC/Cur5 releasate (Figure 6B–J).

### 3.7. Antibacterial Activity

#### 3.7.1. Bacterial Viability Assessment

**Figure 7:**
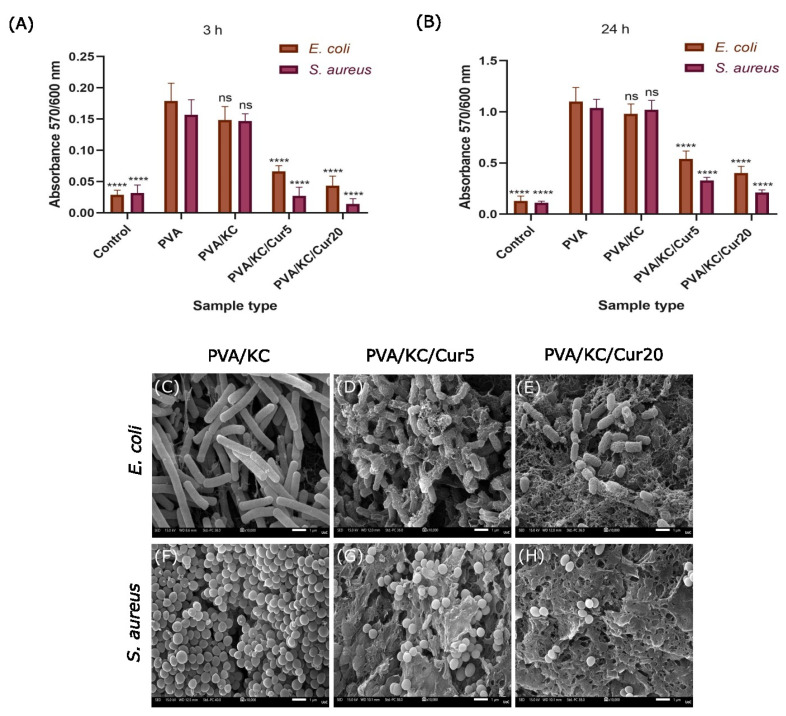
Optical density measurement values of bacteria seeded on the different scaffolds after (**A**) 3 h and (**B**) 24 h. Values are expressed as mean ± standard deviation. Positive control displays the condition with the addition of antibiotics. Statistical analysis was performed among the samples in comparison with PVA membrane (non-significant (ns) *p* > 0.05, **** *p* < 0.0001). Representative scanning electron microscopy images showing the effect of the PVA/KC (control), PVA/KC/Cur5, and PVA/KC/Cur20 membranes on *E. coli* (**C**–**E**) and *S. aureus* (**F**–**H**) after 24 h in culture.

The viability of Gram-positive *S. aureus* and Gram-negative *E. coli* seeded on all scaffold compositions was investigated to confirm the antibacterial properties of the Cur-loaded scaffolds. For this purpose, optical density measurements of the two types of bacteria seeded on the scaffolds were obtained after 3 and 24 h. As shown in Figure 7A,B, both Cur-loaded membranes exhibited significantly higher antibacterial activity against both bacterial types compared with the PVA and PVA/KC membranes at both time points. Both Cur-loaded membranes inhibited bacterial growth, with PVA/KC/Cur20 showing the most pronounced antibacterial effect. After 3 h, no significant difference in the antibacterial efficiency was observed among the Gram-negative *E. coli* and the Gram-positive *S. aureus*. However, after 24 h, the viability was higher in the case of *E. coli* for both the PVA/KC/Cur5 and PVA/KC/Cur20 membranes. No significant difference in bacterial viability was observed between the PVA and PVA/KC membranes.

#### 3.7.2. Visualization of Bacterial Morphology by SEM

To visualize the morphological alterations in Gram-positive *S. aureus* and Gram-negative *E. coli* bacteria cultured on the Cur-loaded membranes, SEM imaging was employed (Figure 7C). The bacterial cell structure was observed after 24 h in culture, and the bacterial morphology was compared with control samples grown on the PVA/KC membrane. The SEM images revealed that both PVA/KC/Cur5 and PVA/KC/Cur20 membranes caused significant disruptions in the normal bacterial morphology, leading to visible membrane damage. These effects were more pronounced in *S. aureus*, where the bacteria displayed more membrane deformation and damage, particularly on the PVA/KC/Cur20 membranes. The images showed that the membranes of *S. aureus* cells were shrunken and damaged, indicating a severe impact on bacterial cell structure. In contrast, *E. coli* bacteria on the Cur-loaded membranes also exhibited morphological alterations, but the damage was less severe than that observed in *S. aureus*. Moreover, the PVA/KC/Cur20 membrane appears to exhibit more severe membrane disruption for both bacteria in comparison with the PVA/KC/Cur5 membrane.

#### 3.7.3. Growth Kinetics of *S. aureus* and *E. coli*

**Figure 8:**
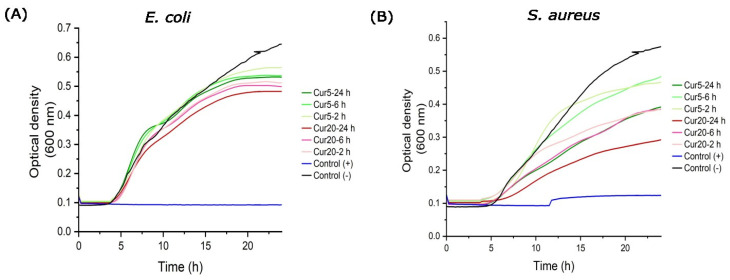
Kinetics of (**A**) *E. coli* and (**B**) *S. aureus* at releasates of PVA/KC/Cur5 and PVA/KC/Cur20 membranes after 2, 6, and 24 h. Optical density measurements were collected for 24 h at 37 °C using a microplate reader at 600 nm and automatically recorded every 15 min. Negative controls (control (–)) were the bacterial suspension in MHB. Positive controls (control (+)) were wells with the bacterial suspension in the presence of augmentin.

The antibacterial activity of the releasates of the Cur-loaded membranes, collected at 2, 6, and 24 h, against *E. coli* and *S. aureus* was evaluated to monitor the bacterial growth kinetics over the course of 24 h (Figure 8). Optical density (OD) measurements were recorded every 15 min to assess the ability of Cur released from the membranes to hinder the growth of bacteria. Both membranes’ releasates consistently demonstrated lower OD values compared with the negative control, indicating the significant antibacterial properties of Cur. Among the two membrane types, the releasates of the PVA/KC/Cur20 membrane showed a higher efficacy at inhibiting bacterial growth at all time points. Moreover, the bacterial inhibition was higher for *S. aureus* than for *E. coli* at all time points, suggesting that *E. coli* is more resistant to Cur than *S. aureus*.

## 4. Discussion

The average fiber diameter of the PVA and PVA/KC fibrous membranes was analyzed using SEM microscopy. While both membranes exhibited similar average fiber diameters, the PVA/KC membrane showed a slightly larger average diameter. This increase in fiber diameter may be attributed to the higher viscosity of the PVA/KC solution at room temperature, which affects the electrospinning process [18]. Moreover, the slight variation in voltage during electrospinning (130 V for the PVA/KC membrane and 140 V for the PVA membrane) could also contribute to the differences in fiber morphology, as lower voltages are known to produce thicker fibers by reducing the elongation forces on the electrospinning jet [25].

TGA analysis of the membrane compositions showed that the decomposition of KC occurs at higher temperatures, indicating that its addition enhances the thermal resistance of the membrane. This delay in weight loss compared with pure PVA can be attributed to the decomposition of the polysaccharide structure of KC, which likely involves the formation of hydrogen bonds between PVA and KC. These interactions create a more stable network, requiring more energy to break down. With the incorporation of Cur, the degradation curves shift further to higher temperatures, suggesting an additional increase in thermal stability. This enhanced stability may be due to the aromatic structure of Cur, which can form π-π interactions and additional hydrogen bonds with the PVA/KC matrix. Moreover, the PVA membrane exhibited the lowest residual mass, suggesting complete decomposition, while PVA/KC, PVA/KC/Cur5, and PVA/KC/Cur20 had higher residual masses. Among the Cur-loaded membranes, thermal resistance increased with the concentration of Cur, likely due to the increased cross-linking.

The hydrophilic properties of the membranes were evaluated using contact angle measurements. The results indicated that the PVA and PVA/KC/Cur20 membranes exhibited the lowest hydrophilicity, while the PVA/KC membrane demonstrated the highest hydrophilicity. The contact angle for the pure PVA membrane was approximately 63°, which aligns with previous studies reporting similar values for electrospun PVA membranes [26]. The significant reduction in the contact angle observed upon the addition of KC to PVA is consistent with findings from other studies, where even a small concentration of KC in electrospun matrices drastically enhanced hydrophilicity. This can be attributed to the abundant hydroxyl and sulfate groups in the KC structure, which can form strong hydrogen bonds with water, significantly increasing the membrane’s water affinity [27,28]. Moreover, the hydrophilicity of the PVA/KC membrane decreased after coating it with Cur via electrospraying. The reduction in hydrophilicity became more pronounced as the Cur concentration increased. This behavior can be attributed to the hydrophobicity of Cur. This is in agreement with other studies, where Cur has been reported to increase the surface hydrophobicity when incorporated into polymer matrices [29].

The swelling behavior of scaffolds plays a critical role in promoting faster wound healing, because scaffolds with swelling capacity aid a moist environment and prevent infections by absorbing wound exudates [30]. Therefore, the swelling capacities of the various scaffold compositions were examined. The PVA/KC membrane exhibited the highest swelling ratio, nearly 1.5-fold higher than that of the pure PVA membrane. This significant increase in swelling can be attributed to the hydrophilic nature of KC. In contrast, the swelling capacity was reduced upon incorporation of Cur into the PVA/KC membranes, with the PVA/KC/Cur20 membrane showing a more pronounced decrease. This decrease can also be linked to the hydrophobic nature of Cur, leading to limited ability of the scaffold to absorb water.

In wound-healing applications, a scaffold must also exhibit a controlled degradation rate. It needs to slowly degrade as the new tissue grows and be gradually resorbed once the wound is healed. The degradation behavior of the membranes followed patterns observed in similar materials, with all membranes displaying a gradual increase in the degradation rate over time. The PVA/KC membrane degraded faster than the PVA membrane, which can be attributed to the enhanced hydrophilicity of KC. The increased water absorption in the PVA/KC membrane likely accelerates hydrolytic degradation. However, the addition of hydrophobic Cur into the PVA/KC membranes slowed down the degradation. This characteristic is advantageous for wound-healing scaffolds, as it allows the Cur-loaded membranes to maintain their mechanical integrity for a longer period and provide support during the stages of wound healing. This controlled degradation, combined with Cur’s inherent bioactive properties, enhances the potential of these membranes as scaffolds for skin tissue engineering.

The cumulative release of Cur from the PVA/KC/Cur5 and PVA/KC/Cur20 fibrous scaffolds over 14 days was also assessed. The higher cumulative release from the PVA/KC/Cur20 membrane may be attributed to the higher initial Cur concentration, which made more Cur available for diffusion. The initial burst release of Cur from both membranes is due to the fact the Cur is attached weakly to the fibrous membrane surface [31]. The initial burst release from both membranes could help prevent inflammation, while the sustained release ensures prolonged delivery of Cur during the critical phases of wound healing.

Moreover, we investigated the effect of Cur-loaded scaffolds on the migration of L929 fibroblasts. It has been reported that Cur may be beneficial for cell migration. The PVA/KC/Cur5 scaffold releasate supported wound healing at the same rate as the control group. This is consistent with other work, where Cur has been reported to enhance migration. In a recent study [32], the effect of Cur-loaded lignin nanoparticles on the migration of L929 skin fibroblast cells was reported. The study demonstrated that the scratch surface area treated with the Cur-loaded nanoparticles resulted in higher wound healing closure compared with the control scratch surface area. In another work, Liao et al. confirmed that the released Cur could enhance the migration of HS68 cells compared with control (medium) and Cur-free PLGA nanofiber membranes [33]. The difference in wound healing rates of the PVA/KC/Cur5 and PVA/KC/Cur20 scaffold releasates can be attributed to the toxicity of DMSO used as a solvent for the dissolution of Cur.

SEM images of cells seeded on the four membranes were obtained on days 3 and 7. The results demonstrated that all the membranes exhibited good biocompatibility and cell attachment. To verify the validity of the SEM findings, a quantitative cell viability assay was employed to assess the cell viability and proliferation. The results showed that the inclusion of KC in the membrane enhanced the cell viability on the PVA/KC membranes. The PVA/KC membrane displayed the highest cell viability rate among the scaffolds, followed by the PVA/KC/Cur5 membrane at all time points. This is in line with previous studies, where KC has been reported to support cell adhesion and proliferation [34]. However, in the case of the PVA/KC/Cur20 membrane, the cell viability declined. This reduction can be attributed to the changes in the membrane’s surface roughness caused by the longer duration of electrospraying. Several research studies have reported that increased surface roughness can inhibit cell viability by hindering cell adhesion [35].

During normal wound healing, the fibroblasts secrete collagen during the proliferation phase. After the secreted collagen forms a strong network through cross-linking, the mechanical strength of the wound is restored. Therefore, collagen secretion by fibroblast cells is crucial for the development of skin tissue and wound healing [36]. The collagen secretion from cells seeded on all four scaffold compositions increased over time, proving the capability of the scaffolds to enable cells to produce collagen and promote wound healing. The PVA/KC membrane displayed a significant increase in collagen secretion compared with the PVA membrane. This is consistent with the cell viability experiment, wherein the PVA/KC membranes exhibited the highest cell viability. It is likely that the highest collagen production is a consequence of a better cell attachment and proliferation of cells on the PVA/KC membrane. However, the presence of Cur decreased the collagen production levels for all days.

Antibacterial experiments were conducted against *E. coli* and *S. aureus* strains. Consistent with previous reports, Cur showed a stronger inhibitory effect against Gram-positive *S. aureus* compared with Gram-negative *E. coli* [37,38]. This higher antibacterial efficacy against Gram-positive bacteria is thought to be due to differences in the structure and composition of the bacterial cell walls. Gram-negative bacteria such as *E. coli* possess an additional outer membrane (OM) composed of lipopolysaccharides, which acts as a barrier and makes them more resistant to many drugs and antibiotics, including Cur. In contrast, Gram-positive bacteria like *S. aureus* have a thicker peptidoglycan layer, but they lack the outer membrane, allowing Cur to more easily penetrate and disrupt their cellular processes [38]. The results of the bacterial viability assessment were in line with the SEM images obtained after the bacterial strains were cultured on the membranes. In addition, the kinetic study confirmed the effect of the Cur concentration on the release rate and bacterial growth of the two strains. The PVA/KC/Cur20 membrane exhibited stronger antibacterial activity against *E. coli* and *S. aureus*, particularly over longer periods, compared with PVA/KC/Cur5. These results are in line with bacterial viability experiments, in which Cur-loaded membranes showed a higher efficacy against *S. aureus* than *E. coli*. A similar disruption in the membrane structure of *E. coli* and *S. aureus* was observed in relevant research reports employing antibacterial chitosan and silver ions [19,20]. The combination of the antibacterial and anti-inflammatory properties of Cur with the PVA/KC electrospun nanofibrous membranes highlights their potential as wound dressings.

## 5. Conclusions

In this study, four types of electrospun membranes, PVA, PVA/KC, PVA/KC/Cur5, and PVA/KC/Cur20, were successfully developed using the electrospinning and electrospraying techniques to identify which composition, i.e., Cur concentration, would provide optimal wound healing, in a fibroblast migration assay, and antimicrobial activity using a Gram-positive and a Gram-negative bacterial strain. Physicochemical characterization of the membranes showed that the addition of KC to the PVA scaffold significantly improved the hydrophilicity and swelling ability as well as the degradation rate of the scaffolds, making the PVA/KC membrane more suitable for tissue engineering compared with the PVA membrane. Additionally, the Cur release data revealed a sustained release profile for both Cur-loaded membranes, which is crucial for long-term therapeutic results. Evaluation of the cell migration study showed that among the two Cur-loaded membranes, PVA/KC/Cur5 facilitated faster cell migration. Moreover, studies of L929 cells on the membranes revealed that PVA/KC membrane supported better cell adhesion, proliferation, and collagen production due to its enhanced hydrophilicity and biocompatibility. However, at higher concentrations, Cur altered the surface roughness of the membranes, leading to reduced cell viability and collagen secretion. The findings from the antibacterial assays demonstrated that both Cur-loaded scaffolds exhibited antibacterial properties against both Gram-positive and Gram-negative bacteria, with PVA/KC/Cur20 exhibiting the highest antibacterial efficiency. These results emphasize the potential of Cur-loaded electrospun membranes as multifunctional wound dressings that both provide antibacterial protection and support tissue regeneration. Future studies could further focus on optimizing the Cur concentration to balance the antimicrobial effect with cell compatibility to improve the overall efficacy of the scaffold as a wound dressing for chronic wounds.

## Figures and Tables

**Table 1:** Designation and composition of the electrospun membrane types.

Designation	Composition
PVA	5% *w*/*v* PVA in water
PVA/KC	5% *w*/*v* PVA in water blended with 1% *w*/*v* KC
PVA/KC/Cur5	Electrospraying of the PVA/KC membranes for 5 min with a 10% (*w*/*v*) Cur in a 1:1 volume ratio of dimethyl sulfoxide and water
PVA/KC/Cur20	Electrospraying of the PVA/KC membranes for 20 min with a 10% (*w*/*v*) Cur in a 1:1 volume ratio of dimethyl sulfoxide and water

## Data Availability

Data are available upon request.
